# Bone marrow mastocytosis associated with primary cutaneous follicle center lymphoma: an unusual case report

**DOI:** 10.1007/s00277-025-06588-4

**Published:** 2025-09-06

**Authors:** Max Vincent John, Ingrid Simonitsch-Klupp, Johannes Griss, Cora Waldstein, Harald Herrmann, Alexander Gaiger, Karoline V. Gleixner, Wolfgang R. Sperr, Peter Valent

**Affiliations:** 1 Department of Internal Medicine I, Division of Hematology and Hemostaseology, Medical University of Vienna, Vienna, Austria; 2 Ludwig Boltzmann Institute for Hematology and Oncology, Medical University of Vienna, Vienna, Austria; 3 Department of Pathology, Medical University of Vienna, Vienna, Austria; 4 Department of Dermatology, Medical University of Vienna, Vienna, Austria; 5 Department of Radiation Oncology, Medical University of Vienna, Vienna, Austria; 6 Department of Internal Medicine I, Division of Hematology & Hemostaseology and Ludwig Boltzmann Institute for Hematology and Oncology, Medical University of Vienna, Waehringer Guertel 18-20, Vienna, A-1090 Austria

**Keywords:** Mastocytosis, PCFCL, AHN, ALN

## Abstract

In a subset of patients with systemic mastocytosis (SM), an associated hematologic neoplasm (AHN) is identified. Most AHN are myeloid neoplasms, whereas lymphoid neoplasms are uncommon. We report on a 70-year-old female patient with bone marrow mastocytosis (BMM) associated with primary cutaneous follicle center lymphoma (PCFCL). Initially, the patient had developed BMM at the age of 61 years. Since then, she suffered from recurrent anaphylaxis and vitiligo. A BM investigation revealed several compact aggregates of MC exhibiting CD25 and the *KIT* mutation D816V. The basal serum tryptase level ranged between 26.0 and 30.9 ng/ml. Nine years after the initial diagnosis of BMM, the patient developed cutaneous nodular reddish lesions on her scalp. Histologic examination excluded mastocytosis and revealed PCFCL. Radiation therapy resulted in regression of cutaneous lesions. This unusual case demonstrates that patients with BMM may develop a lymphoid neoplasm in extramedullary sites, including skin. However, both neoplasms were separable from each other by site and pathology, which would argue for two separate etiologies and against a classical AHN.

**Supplementary Information:**

The online version contains supplementary material available at 10.1007/s00277-025-06588-4.

## Introduction

Mastocytosis is a clonal hematopoietic disease defined by expansion of neoplastic mast cells (MC) in one or more organ-systems [1, 2]. Cutaneous mastocytosis (CM) is largely confined to the skin and typically manifests in childhood. By contrast, systemic mastocytosis (SM) is usually diagnosed in adults [1, 2]. In these cases at least one extra-cutaneous organ is involved and patients may or may not present with cutaneous lesions. The diagnosis SM is usually established by bone marrow (BM) examination and is based on diagnostic criteria provided by the World Health Organization (WHO) and/or international consensus classification (ICC) [1–8]. In most SM patients, the somatic *KIT* mutation D816V is detected in neoplastic cells [1, 2, 5, 6].

Based on the burden of neoplastic MC, involvement of additional lineages and presence or absence of SM-induced organ damage, SM is divided into non-advanced SM and advanced SM (AdvSM) [1–8]. Non-advanced SM includes BM mastocytosis (BMM), indolent SM (ISM) and smoldering SM (SSM) [1–8]. BMM is defined by the absence of skin lesions, a basal serum tryptase level < 125 ng/mL, and absence of criteria defining smoldering or AdvSM [7, 8].

AdvSM encompasses aggressive SM (ASM), SM with an associated hematologic neoplasm (SM-AHN) and MC leukemia (MCL) [1, 2, 5, 6]. The prognosis and survival of these patients depend on the aggressiveness of both, the SM and AHN. Most AHN are myeloid neoplasms, whereas lymphoid neoplasms are very rare [1, 2]. Therefore, the ICC changed the terminology and replaced “AHN” by the term “associated myeloid neoplasms” (“AMN”) thereby excluding lymphoid neoplasms [4–6].

However, although rare, lymphoid AHN have also been described, including multiple myeloma and Non-Hodgkin`s lymphoma [8–10]. Whereas in several of these patients, SM and the lymphoid neoplasm develop in the same organ (usually BM), other patients present with typical SM and an AHN that develops in a different organ-system.

We present a case of BMM in whom a primary cutaneous follicle center lymphoma (PCFCL) developed which would meet criteria of an AHN in the WHO classification but does not qualify as AMN by ICC.

## Case report and results

### Case report

A 61-year-old female patient was seen in August 2015 after a severe episode of systemic anaphylaxis with cardiac arrest. The case history revealed bronchial asthma in early childhood, arterial hypertension, vitiligo, and autoimmune thyroiditis. Because of a “sick sinus syndrome” she received a cardiac pacemaker in 2015. Based on the case history and elevated tryptase a BM examination was performed and revealed BMM. Between 2011 and 2024 the patient suffered from recurrent MC activation-related symptoms, including flushing, urticaria, nausea, diarrhea, and hypotension. Several of these episodes were severe, requiring emergency treatment with adrenalin or even resuscitation. In most events, no inducing trigger was identified. However, some episodes of MC activation occurred after intake of certain medications or food. Subsequent studies revealed an increased total serum IgE (292 kU/l, normal range: 0-100 kU/l). However, no IgE-dependent allergy could be detected. The baseline serum tryptase level was slightly elevated (26 ng/mL). In 2016, a blood test showed normal leukocytes (6.13 G/l) and differential counts, a normal hemoglobin (14.1 g/dl) and a normal platelet count (252 G/l) (Table 1). The C-reactive protein was slightly elevated (0.91 mg/dl; normal range: 0-0.5 mg/dl). All other laboratory parameters were within normal range (Supplementary Table S1).

**Table 1:** Follow up of laboratory parameters

Year	Hb (g/dl)	WBC (G/l)	PLT (G/l)	ALP (U/l)	ß2-microglobulin (mg/l)	LDH (U/l)	Albumin (g/l)	Tryptase (ng/ml)
2016	14.1	6.13	252	91	n.a.	232	n.a.	26.0
2017	13.2	4.16	194	88	n.a.	197	44.0	n.a.
2019	14.0	4.36	205	87	2.03	197	45.8	25.2
2020	13.3	3.39	187	85	2.32	203	44.0	25.0
2021	13.3	3.76	180	85	2.18	173	45.9	30.0
2022	14.0	4.85	207	94	2.17	214	48.1	30.2
2023	13.3	4.15	189	88	n.a.	190	47.0	28.1
2024	12.5	3.87	167	86	2.73	187	45.0	28.7

Abbreviations: Hb, hemoglobin; WBC, white blood count; PLT, platelets; ALP, alkaline phosphatase; LDH, lactate dehydrogenase; U/l, units per liter; g/dl, gram per deciliter; G/l, giga per liter; ng/ml, nanogram per milliliter; n.a., not available

### BM studies and staging investigations

**Fig. 1:**
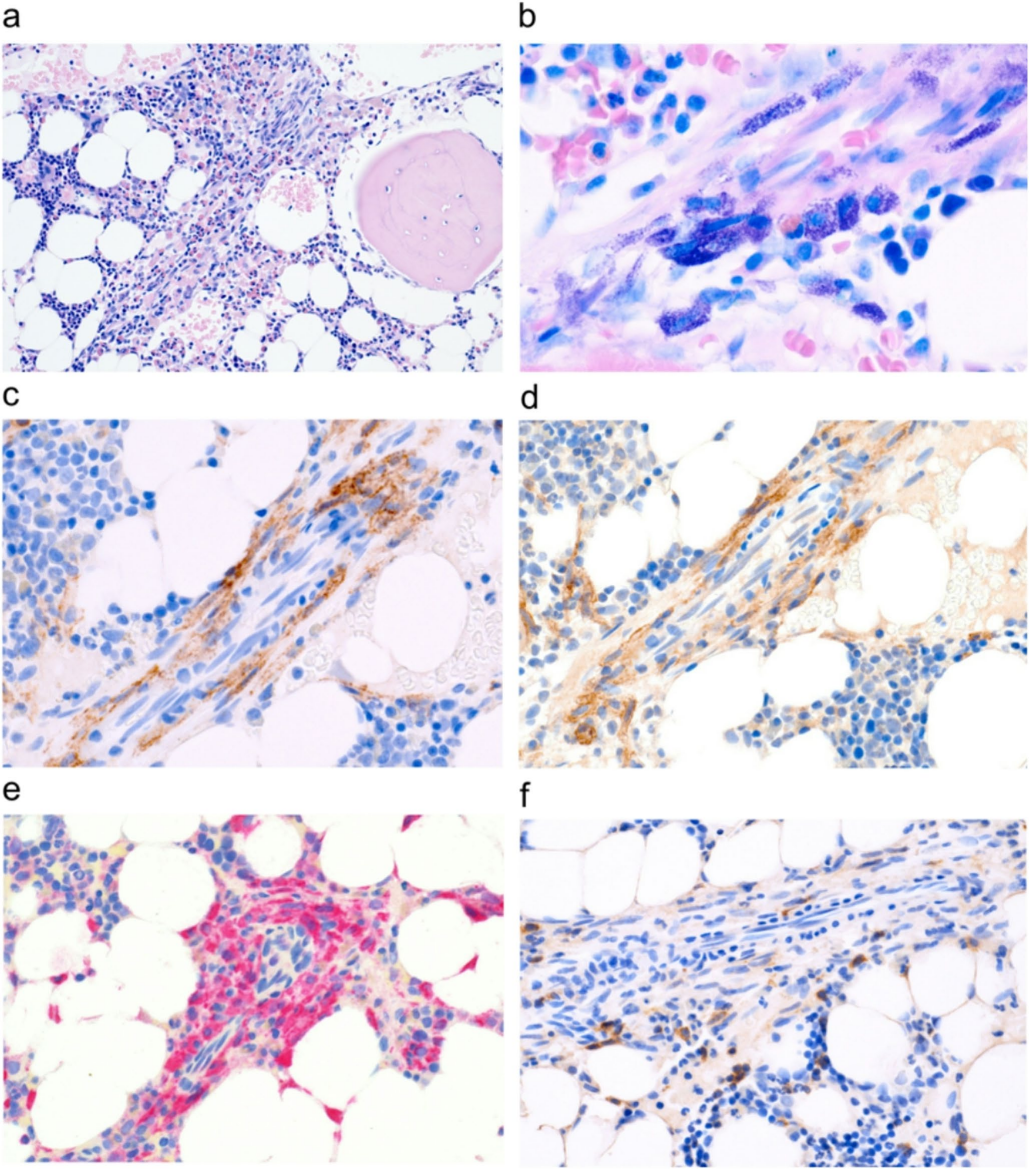
Histologic and immunohistochemical detection of neoplastic mast cells (MC). Bone marrow (BM) was obtained from the iliac crest. After fixation and decalcification, BM sections were stained with H&E (**a**). MC forming compact aggregates accompanied by numerous eosinophilic granulocytes were identified. The Giemsa stain (**b**) shows spindle-shaped granulated MC. Immunohistochemistry revealed expression of CD25 (**c**), CD117 (KIT) (**d**), tryptase (**e**) and weak staining of CD2 (**f**) confirming the presence of SM. Original magnification x100 (**a**), x600 (**b**), x400 (**c-f**)

BM studies included a cytological examination of BM smears, histopathological and immunohistochemical (IHC) investigations of paraffin-embedded BM sections, and molecular studies. IHC revealed the presence of several compact aggregates of atypical tryptase+ and KIT+ MC. These MC accounted for 5% of all nucleated BM cells and stained positive for CD25 and CD117/KIT (Fig. 1). Some MC also stained weakly positive for CD2 and/or CD30. The SM-related *KIT* mutation D816V was identified in BM cells and peripheral blood (PB) leukocytes by qPCR. The remaining BM was normal without signs of myelodysplasia, fibrosis, or an AHN. In staging and grading investigations, no evidence for involvement of the skin or other organs and no signs for AdvSM were found. In particular, no osteolysis, no splenomegaly and no lymphadenopathy were detected. However, bone densitometry revealed osteopenia (T score -1.4). Based on staging- and grading-results, a diagnosis of BMM was established.

### Clinical course and detection of PCFCL

Based on her case history and symptoms the patient was treated with histamine H1 and H2 receptor-antagonists and received an emergency kit including oral corticosteroids and an adrenaline self-injector with advice when and how to use in case of severe anaphylaxis. In addition, she received vitamin D3. Between 2016 and 2024, the clinical course and laboratory parameters were stable and the basal serum tryptase level ranged between 26.0 and 30.9 ng/ml (Table 1, Supplementary Table S1).

**Fig. 2:**
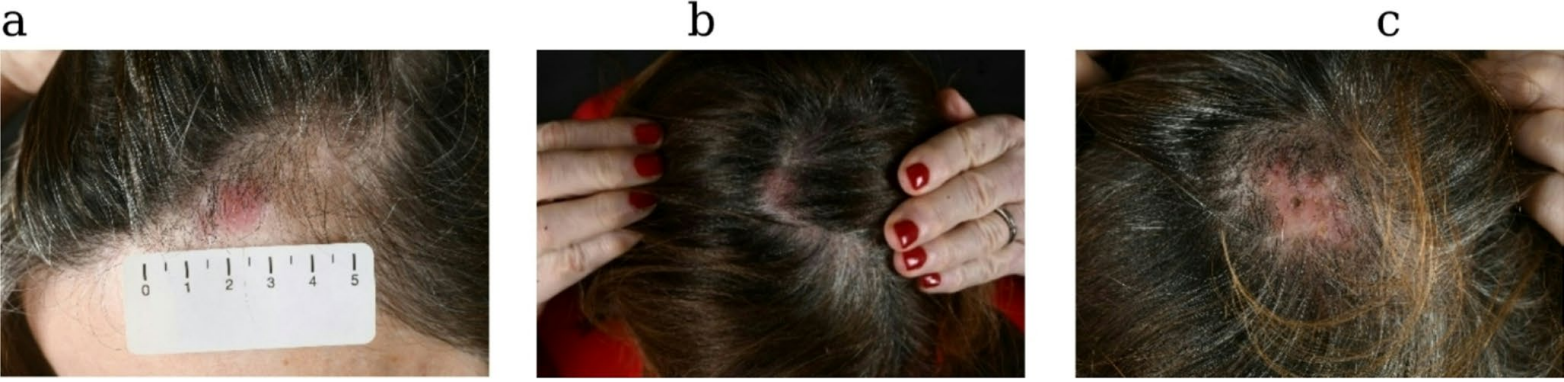
Erythematous cutaneous lesions in the left frontal capillitium (**a**), right parieto-occipital (**b**), and left occipital region (**c**) of the patient.

**Fig. 3:**
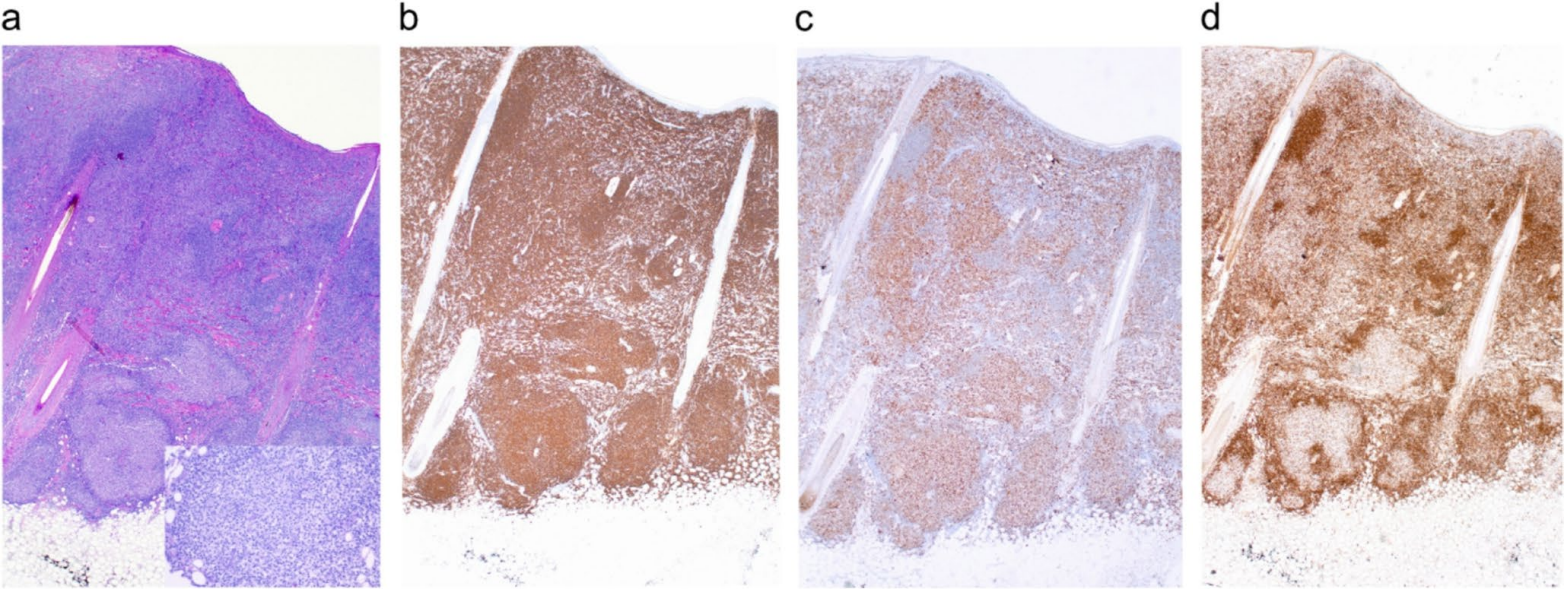
Histology and immunohistology of serial sections of the cutaneous tumor mass. H&E staining (**a**) revealed a dense bottom-heavy dermal atypical lymphoid infiltrate comprising neoplastic germinal-center-like structures composed of mainly small- to medium sized centrocyte-like cells with few centroblasts surrounded by ill-defined mantle zones; follicular polarization is absent (inset). Lesional cells stained positive for CD20 (**b**) and BCL-6 (**c**), but negative for BCL-2 (**d**). Original magnification x 200.

In January 2024, at the age of 70 years, the patient presented with non-scaling erythematous papules and plaques at her forehead skin (left parieto-occipital) measuring 3 cm in diameter and several smaller papules at the right parieto-occipital and left occipital capillitium (Fig. 2). Histological examination revealed a dense and diffuse lymphoid infiltrate displaying a nodular pattern that extended into the subcutaneous adipose tissue. The infiltrating lymphocytes stained positive for CD19, CD20, and CD79a and co-expressed BCL-6, but did not exhibit BCL-2. The proliferation rate (Ki-67-staining) within follicles was approximately 25%. (Fig. 3). Staging investigations, including a positron emission computed tomography (PET-CT), did not reveal extracutaneous lesions and thus confirmed the presence of a primary cutaneous lymphoma (PCL). Based on these results and phenotype of neoplastic cells, the diagnosis of PCFCL was established. In the context of mastocytosis, the diagnosis changed from SM (BMM) to SM-AHN, subtype BMM-PCFCL as defined by WHO criteria. During the next few months, the clinical course was stable. In September 2024, a control CT did not reveal new lymphoma lesions in the skin or other organs.

### Treatment of PCFCL

Radiation therapy is considered standard treatment in PCFCL patients, resulting in a high remission rate [10, 11]. Therefore, the patient received local radiotherapy using superficial orthovoltage energy photons (100 kilovolt). The clinical target area included a safety margin of 10 mm to encompass and treat subclinical disease. The total radiation dose of 24 Gy was applied in 12 fractions. In response to this therapy, skin lesions decreased in size, and after 3 months no remaining skin lesions were detected.

## Discussion

In most patients with SM-AHN, the SM component and AHN are clearly separable by histology, phenotyping and molecular aberration-profiles. In addition, SM and the AHN often differ in aggressiveness and clinical course [1, 2, 8]. While patients with BMM or ISM without an AHN have an almost normal life expectancy, survival in patients with AdvSM including SM-AHN, is usually dismal, especially when the AHN is an advanced disease [1, 2, 8]. In our patient, BMM was initially diagnosed, and during follow-up a PCFCL was detected, changing the diagnosis to BMM-PCFCL according to the WHO classification. Although similar rare SM cases with cutaneous lymphomas have been described [12] our report is the first case of BMM-PCFCL.

Whereas myeloid neoplasms are common in SM-AHN patients, associated lymphoid neoplasms are very rare [1, 2, 9–14]. Therefore, the ICC changed the term AHN to “associated myeloid neoplasms” (SM-AMN) thereby excluding lymphoid neoplasms from this group [4–6]. However, in some cases with SM, an associated lymphoid neoplasm is indeed diagnosed [9–14]. In a recent attempt to harmonize the WHO and ICC, the term “associated lymphoid neoplasm” (ALN) was proposed for such cases [15]. In this harmonized classification, our patient would also qualify as SM-ALN. However, several features and findings argue against a common origin of both neoplasms. First, the two neoplasms developed in different organ systems and exhibited completely separable phenotypes. In addition, the PCFCL developed long (9 years) after the initial diagnosis of BMM. Unfortunately, we were not able to recover enough lymphoma cells to study molecular lesions in the PCFCL. However, based on the available literature, we assume that the lymphoma cells did not express KIT D816V, which is also supported by the fact that the “bystander MC” in the skin lesion did neither reveal atypical morphology or CD25 expression.

In the updated WHO classification, BMM is a separate category of SM defined by SM criteria, absence of typical skin lesions, absence of B- and C-Findings, and a tryptase level < 125 ng/ml [7, 8, 15]. BMM is also known to bear a high risk of anaphylaxis [7]. In our case, “BMM criteria” were fulfilled and the patient suffered from recurrent anaphylaxis.

In the ICC proposal, our patient was classified as ISM with unrelated PCFCL. However, we cannot exclude that there was a clonal or functional relationship between BMM and PCFCL. First, a very immature totipotent neoplastic hematopoietic stem cell may initially have seeded the BM and only later populated the skin; during stem cell evolution and homing to skin, some of these cells may have switched to the lymphoid lineage. An alternative possibility would be that BMM and/or PCFCL cells produced growth-stimulating cytokines that facilitated the evolution of the other neoplasm. However, this possibility seems unlikely as the two neoplasms developed in different (distant) organs and at different time points.

Most lymphoid neoplasms described in SM-AHN developed in internal organs [1, 2, 9]. However, primary cutaneous lymphomas have also been described in SM patients [10–14]. Such skin lymphomas include follicular B-cell lymphoma and marginal zone B-cell lymphoma (Supplementary Table S2) [10–14]. Our case was diagnosed as BMM with concomitant PCFCL (BMM-PCFCL).

## Supplementary Information

Below is the link to the electronic supplementary material.

Supplementary Material 1 (DOCX. 33.3 KB)

## Data Availability

No datasets were generated or analysed during the current study.

## Abbreviations

AdvSM: Advanced systemic mastocytosis
AHN: Associated hematologic neoplasm
ALN: Associated lymphoid neoplasm
ALP: Alkaline phosphatase
AMN: Associated myeloid neoplasm
ASM: Aggressive systemic mastocytosis
BM: Bone marrow
BCL-6: B-cell lymphoma 6 protein
CD: Cluster of differentiation
Ki-67: Ki-67 protein
BMM: Bone marrow mastocytosis
G/l: Giga per liter
g/d1: Grams per deciliter
Hb: hemoglobin
H&E: hematoxylin and eosin
IgE: Immunoglobulin E
ICC: International Consensus Classification
IHC: Immunohistochemistry
ISM: Indolent systemic mastocytosis
kU/l: Kilo unit per liter
LDH: Lactate dehydrogenase
MC: Mast cell
PB: Peripheral blood
PCFCL: Primary cutaneous follicle center lymphoma
PLT: Platelets
SM: Systemic mastocytosis
SM-AHN: Systemic mastocytosis with an associated hematologic neoplasm
SM-ALN: Systemic mastocytosis with an associated lymphoid neoplasm
SSM: Smoldering systemic mastocytosis
WHO: World Health Organization
