# Supplementary material for: Bone marrow mastocytosis associated with primary cutaneous follicle center lymphoma: an unusual case report
Source: Ann Hematol. 2025 Sep 6;104(10):5543–8. doi: 10.1007/s00277-025-06588-4 (PMC12619747; doi:10.1007/s00277-025-06588-4)
Supplement: Supplementary file 1 — Supplementary Material 1 (DOCX. 33.3 KB) [file 277_2025_6588_MOESM1_ESM.docx]

**John et al., Supplementary to:**

**Bone Marrow Mastocytosis associated with Primary Cutaneous**

**Follicle Center Lymphoma: An Unusual Case Report**

Max Vincent John^1,2^, Ingrid Simonitsch-Klupp^3^, Johannes Griss^4^, Cora Waldstein^5^, Harald Herrmann^2,5^, Alexander Gaiger^1,2^, Karoline V. Gleixner^1,2^,

Wolfgang R. Sperr^1,2^, Peter Valent^1,2^

^1^Department of Internal Medicine I, Division of Hematology and Hemostaseology, Medical University of Vienna, Austria. ^2^Ludwig Boltzmann Institute for Hematology and Oncology, Medical University of Vienna, Austria. ^3^Department of Pathology, Medical University of Vienna, Austria. ^4^Department of Dermatology, Medical University of Vienna, Austria. ^5^Department of Radiation Oncology, Medical University of Vienna, Austria.

Running Title: Systemic mastocytosis associated with lymphoid neoplasms

Correspondence to:

Peter Valent, M.D.

Department of Internal Medicine I

Division of Hematology & Hemostaseology and

Ludwig Boltzmann Institute for Hematology and Oncology

Medical University of Vienna,

Waehringer Guertel 18-20

A-1090 Vienna, Austria

Phone: + 43 1 40400 60850

[E-mail: peter.valent@meduniwien.ac.at](mailto:E-mail:%20peter.valent@meduniwien.ac.at)

**Supplementary (S) Tables**

**Table S1**

**Laboratory parameters at the time of diagnosis of BMM (2016) and**

**at the time of diagnosis of PCFCL (BMM-PCFCL) (2024)**

|  | **Diagnosis - Year** | |  |
| --- | --- | --- | --- |
| **Parameter** | **BMM - 2016** | **BMM-PCFCL - 2024** | **Normal range of lab parameter** |
| Erythrocytes | 4.6 T/l | 4.3 T/l | 3.8-5.3 T/l |
| Hb | 14.1 g/dl | 13.0 g/dl | 12.0-16.0 g/dl |
| Hematocrit | 39.1 % | 38.1 % | 35.0-47.0 % |
| MCV | 84.4 fl | 89.2 fl | 78.0-98.0 fl |
| MCH | 30.5 pg | 30.4 pg | 27.0-33.0 pg |
| PLT | 252 G/l | 172 G/l | 150-350 G/l |
| WBC | 6.13 G/l | 3.47 G/l | 4.0-10.0 G/l |
| ANC | 53.1 % | 56.8 % | 50-75 % |
| Lymphocytes | 36.1 % | 31.1 % | 25.0-40.0 % |
| Monocytes | 8.2 % | 10.1 % | 0.0-12.0 % |
| Eosinophils | 1.8 % | 1.4 % | 0.0-4.0 % |
| Basophils | 0.8 % | 0.6 % | 0.0-1.0 % |
| Na^+^ | 140 mmol/l | 144 mmol/l | 136-145 mmol/l |
| K^+^ | 4.24 mmol/l | 4.73 mmol/l | 3.5-5.1 mmol/l |
| Cl^-^ | 102 mmol/l | 107 mmol/l | 98-107 mmol/l |
| Ca^2+^ | 2.32 mmol/l | 2.35 mmol/l | 2.20-2.55 mmol/l |
| Creatinine | 0.71 mg/dl | 0.76 mg/dl | 0.50-0.90 mg/dl |
| Bilirubin | 0.57 mg/dl | 0.50 mg/dl | 0.0-1.2 mg/dl |
| Lipase | 26 U/l | 41 U/l | 13-60 U/l |
| ALP | 91 U/l | 87 U/l | 35-105 U/l |
| ALAT | 23 U/l | 13 U/l | <35 U/l |
| ASAT | 27 U/l | 19 U/l | <35 U/l |
| g-GT | 14 U/l | 14 U/l | <40 U/l |
| LDH | 232 U/l | 206 U/l | <250 U/l |
| CRP | 0.91 mg/dl | 0.91 mg/dL | < 0.5 mg/dl |
| Tryptase | 26.0 ng/ml | 28.7 ng/ml | <11.4 ng/ml |
| IgE | 292 kU/l | 97.6 kU/l | <100 kU/l |

Abbreviations: BMM, bone marrow mastocytosis; PCFCL, primary cutaneous follicle center lymphoma; Hb, hemoglobin; MCV, mean corpuscular volume; fl, femtoliter; pg, picogram; T/l, terra per liter; G/l, giga per liter; g/dl, gram per deciliter; mg/dl, milligram per deciliter; ng/ml, nanogram per milliliter; kU/l, kilounits per liter; MCH, mean corpuscular hemoglobin; ANC, absolute neutrophil count; WBC, white blood count; %, percentage; Na^+^, sodium; K^+^, potassium; Cl^-^, chloride; Ca^2+^, calcium; ASAT, aspartate aminotransferase; ALAT, alanine aminotransferase; g-GT, gamma-glutamyl transferase; PLT, platelets; ALP, alkaline phosphatase; LDH, lactate dehydrogenase; CRP, C-reactive protein; IgE, Immunoglobulin E.

**Table S2**

**Reported cases of primary cutaneous lymphomas (PCL) in mastocytosis patients**

**---------------------------------------------------------------------------------------------------------------------------------------------------------------------------------------------**

**Key clinical, pathological and laboratory findings in the reported patients**

**---------------------------------------------------------------------------------------------------------------------------------------------------------------------------------------------------------------**

serum time from histological

Author and sex age type of tryptase SM to PCL PCL reported

Publication f/m years mastocytosis KIT D816V ng/mL years subtype* phenotype

**----------------------------------------------------------------------------------------------------------------------------------------------------------------------------------------------------------------**

Kincaid et al. 2023 [S1] m 35 CM n.r. n.r. 7 cutaneous follicular B-cell lymphoma CD20, CD21, BCL2, BCL-6

Meyer et al. 2013 [S2] m 41 ISM with CI + 115 3 cutaneous follicular B-cell lymphoma CD10, CD20, BCL-6

Lee et al. 2021 [S3] m 53 CM n.r. n.r. 7 PCMZL CD20, BCL-2, BCL-6

Günay et al. 2022 [S4] f 31 ISM with CI + 22 3 PCMZL CD20, BCL-2

John et al.

current manuscript f 61 BMM** + 28.7 9 PCFCL CD19, CD20, CD79a, BCL-6

**----------------------------------------------------------------------------------------------------------------------------------------------------------------**

*Histological subtypes are shown according to the terminology reported at the time of publication.

**Note that in this case, but not in other patients previously reported, BMM was diagnosed together with a cutaneous lymphoma.

Abbreviations: PCL, primary cutaneous lymphoma; f, female, m, male; PCMZL, primary cutaneous marginal zone lymphoma; PCFCL, primary cutaneous follicle center lymphoma; CM, cutaneous mastocytosis; SM, systemic mastocytosis; ISM, indolent systemic mastocytosis; CI, cutaneous involvement; BMM, bone marrow mastocytosis; ng/ml, nanograms per milliliter; +, mutation detected, - no mutation detected, n.r., not reported.

**Supplementary References**

S1. Kincaid CM, Phong C, Arnold JD, et al. (2023) Primary cutaneous lymphoma in a patient with mastocytosis: Is there an association? JAAD Case Rep 36:70–72. https://doi.org/10.1016/j.jdcr.2023.03.015

S2. Meyer KM, Landthaler M, Hafner C, Geissinger E (2013) Systemic mastocytosis associated with cutaneous B-cell lymphoma. Br J Dermatol 169:1165–1167. https://doi.org/10.1111/bjd.12471

S3. Lee HY, Lee JS, Koo DW (2021) A case report of primary cutaneous marginal zone B-cell lymphoma with mastocytosis. SAGE Open Med case Rep 9:2050313X211042527. https://doi.org/10.1177/2050313X211042527

S4. Günay MB, Büyükbabani N, Yavuz AS, Yılmaz I, Baykal C (2022) Association of systemic mastocytosis with primary cutaneous marginal zone lymphoma; first case. Journal of the European Academy of Dermatology and Venereology: JEADV, *36*(4), e275–e276. https://doi.org/10.1111/jdv.17797
